# Association between CVAI-defined body composition phenotypes and prediabetes outcomes in Chinese adults undergoing health check-ups: a cross-sectional study

**DOI:** 10.3389/fnut.2026.1784292

**Published:** 2026-05-14

**Authors:** Ren Lin, Wang Jiang, Dan Wang, Lijuan Xu, Yaling Du

**Affiliations:** 1 Physical Examination Center, Renmin Hospital of Wuhan University, Wuhan, China; 2 School of Management, Hubei University of Chinese Medicine, Wuhan, China; 3 Department of Public Health, The First Affiliated Hospital of Shihezi University, Shihezi, China

**Keywords:** body-composition phenotype, Chinese visceral adiposity index, low relative muscle mass, prediabetes, visceral obesity

## Abstract

**Background:**

Prediabetes presents a critical opportunity to prevent type 2 diabetes and its complications. Both visceral adiposity and low skeletal muscle mass are closely linked to insulin resistance; however, it remains unclear whether body composition phenotypes—defined by relative muscle mass and the Chinese visceral adiposity index (CVAI), which is a validated measure of visceral fat accumulation in Chinese adults—are differentially associated with specific prediabetes outcomes. Therefore, we examined the associations between CVAI-defined body composition phenotypes and impaired fasting glucose (IFG), elevated HbA1c levels, and composite prediabetes and compared the discriminatory performance of the CVAI with that of BMI and the waist-to-height ratio (WHtR).

**Methods:**

Data obtained from 3,905 adults who attended a health check-up center in 2024 were analyzed. Participants were classified into four body composition phenotypes: Normal, low relative muscle mass alone, visceral obesity alone, and combined low relative muscle mass with visceral obesity. Associations with each prediabetes outcome were estimated using logistic regression to obtain odds ratios (ORs) and 95% confidence intervals (CIs) across three models: Model 1, unadjusted; Model 2, adjusted for age and sex; and Model 3, further adjusted for systolic blood pressure, diastolic blood pressure, low-density lipoprotein cholesterol (LDL-C), alanine aminotransferase (ALT), and estimated glomerular filtration rate (eGFR). The discriminatory performance of the CVAI, BMI, and waist-to-height ratio was evaluated using receiver operating characteristic curves (AUCs), with between-index comparisons conducted using the DeLong test.

**Results:**

Among the 3,905 adults, the phenotype distribution was as follows: 68.0% had a normal phenotype, 6.9% had low relative muscle mass alone, 11.9% had visceral obesity alone, and 13.2% had a combination of low relative muscle mass and visceral obesity. After adjusting for age and sex, the phenotype combining low relative muscle mass with visceral obesity showed the strongest associations with IFG, elevated HbA1c levels, and composite prediabetes (ORs 2.42–2.84). In contrast, obesity alone remained associated with elevated HbA1c levels and composite prediabetes, and low relative muscle mass alone was not independently associated. These findings remained materially unchanged in the extended confounder-adjusted model.

**Conclusion:**

The phenotype characterized by low relative muscle mass combined with high CVAI-defined visceral adiposity was most strongly associated with impaired fasting glucose, elevated HbA1c levels, and composite prediabetes, whereas low relative muscle mass alone showed no independent association after adjustment. The CVAI also demonstrated better discrimination of abnormal glycemia compared to BMI and the waist-to-height ratio.

## Introduction

Prediabetes is an intermediate metabolic state that lies between normal glucose regulation and type 2 diabetes mellitus (T2DM). It represents a critical period for preventing diabetes-related complications. Globally, intermediate hyperglycemia affects over one billion adults (aged 20–79 years) and is projected to become even more common by 2050 (1). Large nationally representative surveys have shown that the prevalence of prediabetes among Chinese adults is approximately 35–40%, which exceeds the prevalence of diabetes itself. This rate is increasing in conjunction with rapid population aging and urbanization (2–4). These trends place a significant burden on the healthcare system, emphasizing the need for simple tools to identify individuals at high risk of glycemic deterioration during the early, reversible stages (5–7).

Obesity, particularly visceral adiposity, plays a central role in the development of insulin resistance and impaired glucose metabolism. Excess visceral fat increases the release of free fatty acids, induces low-grade inflammation, and causes ectopic fat deposition in the liver and skeletal muscle, impairing insulin signaling and glucose disposal (8, 9). Visceral obesity may also accelerate muscle loss through chronic inflammation, physical inactivity, and ectopic fat infiltration of skeletal muscle, thereby linking adiposity to sarcopenia (9–12). In parallel, sarcopenia—characterized by reduced skeletal muscle mass, strength, and/or physical performance—further aggravates glucose dysregulation, as skeletal muscle is the primary site of postprandial glucose uptake (13). When obesity and sarcopenia coexist, the metabolic risk of sarcopenic obesity (SO) is amplified beyond the individual risks of each condition. SO has been recognized as a high-risk phenotype for cardiometabolic complications (13, 14).

Accumulating evidence indicates that sarcopenic obesity is linked to dysglycemia through adulthood. Individuals with prediabetes or diabetes tend to exhibit lower skeletal muscle mass and poorer physical function, and prediabetes has been associated with an increased risk of sarcopenia (15, 16). Moreover, when low muscle mass coexists with obesity, the risk of prediabetes, particularly impaired glucose tolerance, is substantially higher than when either condition occurs alone (17).

However, previous studies have varied substantially in how they define sarcopenic obesity (13, 18, 19). Importantly, the “obesity” component has often been defined using BMI or waist circumference, which may not adequately capture visceral adiposity (20, 21). In addition, many studies have focused on type 2 diabetes rather than distinct prediabetes phenotypes (9, 22). Therefore, incorporating visceral adiposity indices can better capture metabolically relevant obesity than BMI-based definitions when evaluating early dysglycemia.

The Chinese visceral adiposity index (CVAI) is a composite measure that incorporates age, BMI, waist circumference, triglycerides, and high-density lipoprotein cholesterol (HDL-C). It is specifically designed to estimate visceral fat accumulation in Chinese adults (20). Prospective data show that the CVAI predicts the onset of prediabetes and T2DM more accurately than traditional obesity indices, such as BMI and the waist-to-height ratio (WHtR) (23). Despite accumulating evidence, few studies have simultaneously examined the relationship between low relative muscle mass and visceral adiposity, as defined by the CVAI, in relation to various prediabetes phenotypes in Chinese adults.

To address these gaps, we performed a cross-sectional analysis of a large Chinese health check-up cohort and adopted a four-phenotype framework commonly used in previous studies to disentangle the independent and joint contributions of low relative muscle mass and excess visceral adiposity. The four phenotypes were defined as normal, low relative muscle mass alone, visceral obesity alone, and combined low relative muscle mass with visceral obesity (24–26).

Specifically, we evaluated the associations between these phenotypes—defined using sex-specific percentiles of the appendicular muscle mass-to-weight percentage (ASM/Wt) and the CVAI—and impaired fasting glucose, elevated HbA1c levels, and composite prediabetes. Additionally, we examined whether the CVAI provides better discrimination of abnormal glycemia compared to BMI and the waist-to-height ratio. We hypothesized that the combined phenotype, characterized by low relative muscle mass and visceral obesity, would show the strongest association with prediabetes and that the CVAI would outperform traditional anthropometric indices.

## Methods

### Study design and population

We performed a retrospective cross-sectional study using de-identified data from adults who attended the health check-up center at Renmin Hospital of Wuhan University, Hubei, China, between 1 January and 31 December 2024. Eligible participants were aged ≥18 years and underwent a standardized examination including anthropometric measurements, bioelectrical impedance analysis (BIA), and fasting laboratory tests. This study was carried out following the STROBE statement for cross-sectional studies. The protocol was approved by the Clinical Research Ethics Committee of Renmin Hospital of Wuhan University (Approval No. WDRY2025-K171) and was conducted in accordance with the Declaration of Helsinki.

Participants were excluded if they (1) lacked waist circumference or BIA-derived body composition data, (2) lacked fasting plasma glucose (FPG) or HbA1c measurements, (3) reported a history of diabetes or current use of glucose-lowering medications or insulin, (4) met laboratory criteria for diabetes (FPG ≥ 7.0 mmol/L or HbA1c ≥ 6.5%), or (5) lacked age or sex information. We included all eligible adults during the study period; no *a priori* sample-size calculation was performed. After exclusions (through complete-case analysis), 3,905 participants were included in the final analytic sample.

### Assessment of prediabetes

After an overnight fast of at least 8 h, venous blood samples were collected. Fasting plasma glucose (FPG) levels were measured using an enzymatic (hexokinase) assay, and HbA1c levels were measured by high-performance liquid chromatography in the hospital’s central laboratory, which participates in external quality-assurance programs. The specific analyzers, manufacturers, and quality-control procedures for all biochemical measurements are summarized in Supplementary Table S1. Prediabetes was defined by the American Diabetes Association (ADA) Standards of Care in Diabetes-2024 as having impaired fasting glucose (IFG; FPG 5.6–6.9 mmol/L) and/or elevated HbA1c levels (5.7–6.4%). We examined three outcomes: IFG, elevated HbA1c, and composite prediabetes (which reflects the presence of either IFG and/or elevated HbA1c levels).

### Assessment of body composition, low relative muscle mass, and visceral obesity phenotypes

Body composition was assessed using multifrequency segmental bioelectrical impedance analysis (BIA) while participants were in the standing position under standardized conditions (details of the device model and measurement protocol are provided in Supplementary Table S1). Appendicular skeletal muscle mass (ASM, in kg) was calculated as the sum of lean mass in both the arms and legs; appendicular skeletal muscle mass index (ASMI, kg/m^2^) was calculated as ASM/height^2^. Relative muscle mass was expressed as ASM-to-weight percentage (ASM/Wt, %) = ASM (kg)/body weight (kg) × 100. Low relative muscle mass was defined as ASM/Wt below the age- and sex-specific 20th percentile within three age strata (18–39, 40–59, and ≥60 years). This 20th-percentile threshold was selected in accordance with previous studies on relative muscle mass and cardiometabolic risk (27–29).

Given that this mass-based definition does not incorporate muscle strength or physical performance, we used the term “low relative muscle mass” throughout the manuscript rather than “sarcopenia” in a diagnostic sense.

Visceral adiposity was estimated using the Chinese visceral adiposity index (CVAI), which is calculated from age, BMI, waist circumference, triglycerides, and HDL-C using sex-specific equations developed and validated for Chinese adults (23). Visceral obesity was defined as the CVAI at or above the age- and sex-specific 75th percentile within the same three age strata (30, 31); the corresponding cutoffs are provided in Supplementary Table S2.

Participants were categorized into four mutually exclusive body composition phenotypes: (1) normal (neither low relative muscle mass nor visceral obesity), (2) low relative muscle mass alone (low relative muscle mass without visceral obesity), (3) visceral obesity alone (visceral obesity without low relative muscle mass), and (4) combined low relative muscle mass with visceral obesity (presence of both low relative muscle mass and visceral obesity).

### Covariates and other measurements

Height and weight were measured using standardized protocols, and BMI was calculated as weight (kg)/height^2^ (m^2^). Waist circumference was measured at the midpoint between the lower rib margin and the iliac crest at the end of a gentle expiration. The WHtR was calculated as waist circumference (cm)/height (cm). Resting blood pressure was measured in the seated position after at least 5 min of rest using an automated oscillometric sphygmomanometer. Detailed information on laboratory assays, device models, and quality-control procedures is provided in Supplementary Table S1.

### Statistical analysis

Statistical analysis was performed using Stata 16.0 (StataCorp, College Station, TX, USA). Continuous variables were assessed for their distributional characteristics and are presented as mean ± SD when they are approximately normally distributed. Skewed variables (e.g., triglycerides) were presented as medians (interquartile ranges) and were natural log-transformed when included as continuous variables in the regression analyses. Categorical variables were presented as counts and percentages.

Baseline characteristics were compared across the four phenotypes using one-way analysis of variance (ANOVA) for approximately normally distributed continuous variables. The Kruskal–Wallis test was used for skewed continuous variables (e.g., triglycerides), and *χ*^2^ tests were used for categorical variables.

Pearson correlation coefficients were used to assess the pairwise associations between BMI, WHtR, and CVAI. The ROC curves were constructed to evaluate and compare the ability of BMI, WHtR, and CVAI to discriminate composite prediabetes. AUC values were compared using the DeLong test.

Logistic regression was used to assess the associations between body composition phenotypes and prediabetes outcomes, with results reported as odds ratios (ORs) and 95% confidence intervals (CIs). For each outcome, we fitted three models: Model 1 (unadjusted), Model 2 (adjusted for age and sex), and Model 3 (further adjusted for systolic blood pressure, diastolic blood pressure, low-density lipoprotein cholesterol (LDL-C), ALT, and eGFR). Model 3 was specified as an extended confounder-adjusted analysis and was based on a complete-case sample because of missing covariate data. Linear trends across phenotypes were tested by modeling phenotype category as an ordinal variable in regression models. Model fit was assessed using McFadden’s pseudo-*R*^2^.

All *p*-values were two-sided, and a *p*-value of <0.05 was considered statistically significant.

## Results

### Participant characteristics

**Figure 1:**
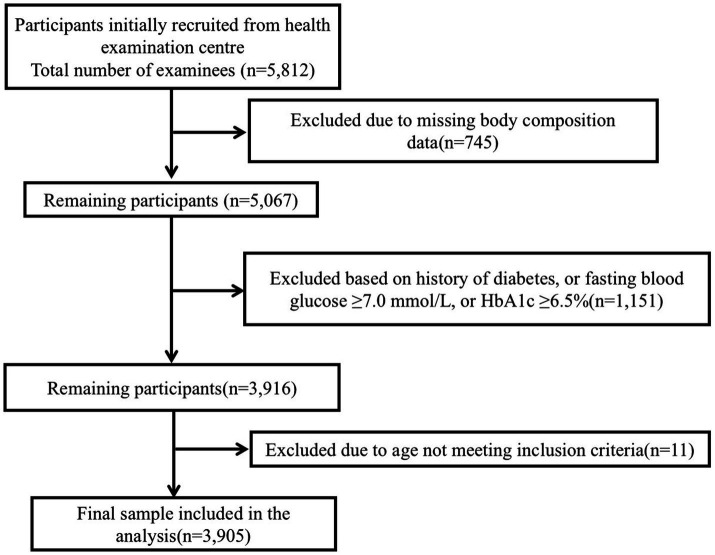
Flow chart of participant selection.

A total of 3,905 adults were included in the analysis [mean (SD) age, 46.4 (14.9) years] (Figure 1). Using age- and sex-specific percentiles of ASM/Wt and the CVAI within three age strata (18–39, 40–59, and ≥60 years), participants were categorized into four phenotypes: Normal (*n* = 2,657; 68.0%), low relative muscle mass alone (*n* = 269; 6.9%), visceral obesity alone (*n* = 465; 11.9%), and combined low relative muscle mass with visceral obesity (*n* = 514; 13.2%). The prevalence of composite prediabetes (IFG and/or elevated HbA1c) increased monotonically across phenotypes (53.6, 62.5, 69.9, and 71.2%, respectively; *p* for trend <0.001).

The BMI, WHtR, and CVAI were positively correlated with one another (all *p* < 0.001), with the strongest correlation observed between the WHtR and CVAI (*r* = 0.892) (Table 1).

**Table 1:** Pairwise correlations between BMI, WHtR, and CVAI.

Pearson correlation coefficient	BMI	WHtR	CVAI
BMI	1.000		
WHtR	0.8495*	1.000	
CVAI	0.7871*	0.8915*	1.000

All correlations were significant at *p* < 0.001. BMI, body mass index; WHtR, waist-to-height ratio; CVAI, Chinese visceral adiposity index. *p < 0.001.

**Figure 2:**
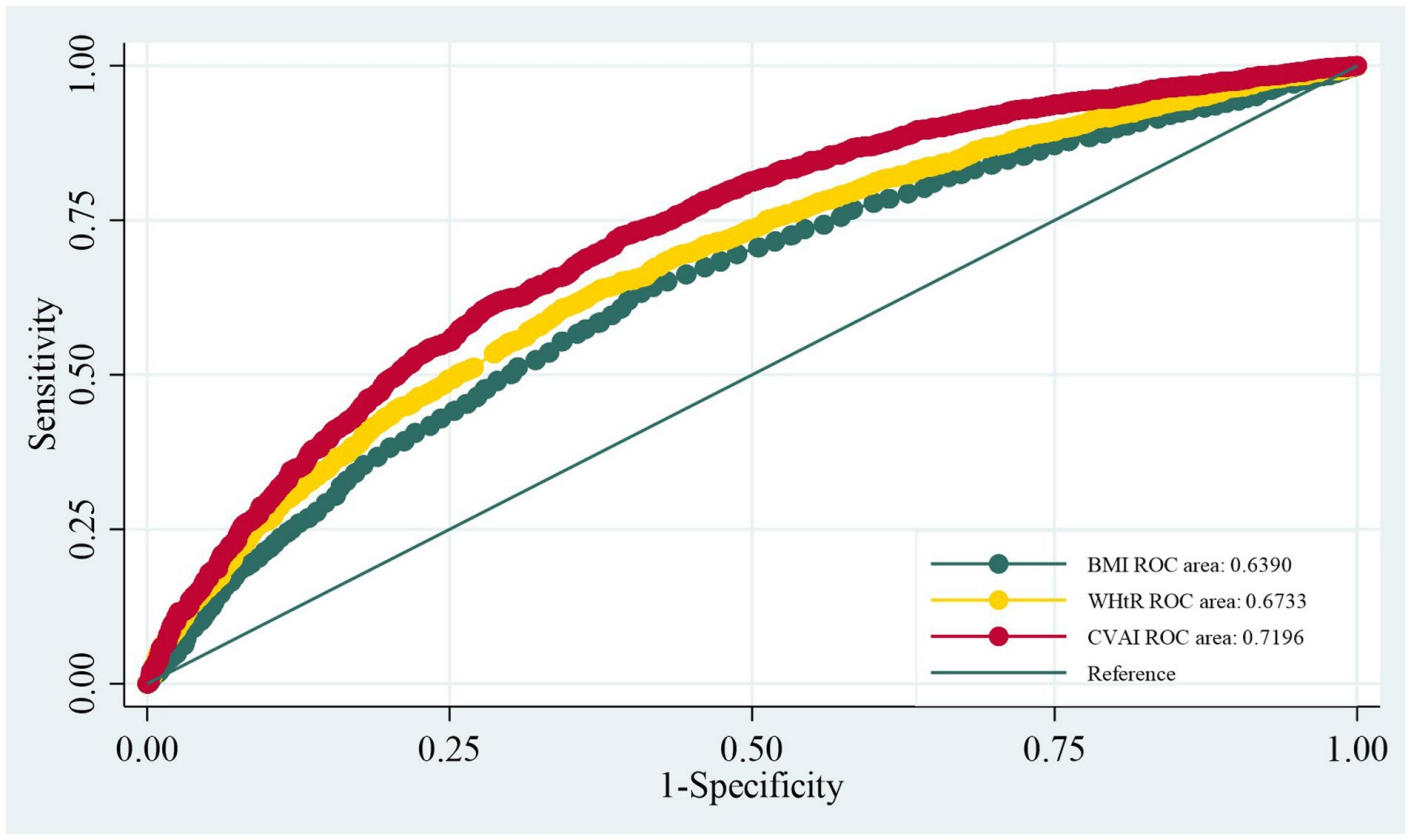
ROC curves for BMI, WHtR and CVAI.

In ROC analyses, the CVAI showed the highest discrimination for composite prediabetes (AUC = 0.720), outperforming BMI (AUC = 0.639) and WHtR (AUC = 0.673). The differences between the indices were significant according to the DeLong test (*χ*^2^ = 196.20, *p* < 0.001) (Figure 2).

### Metabolic and body composition profiles across phenotypes

**Figure 3:**
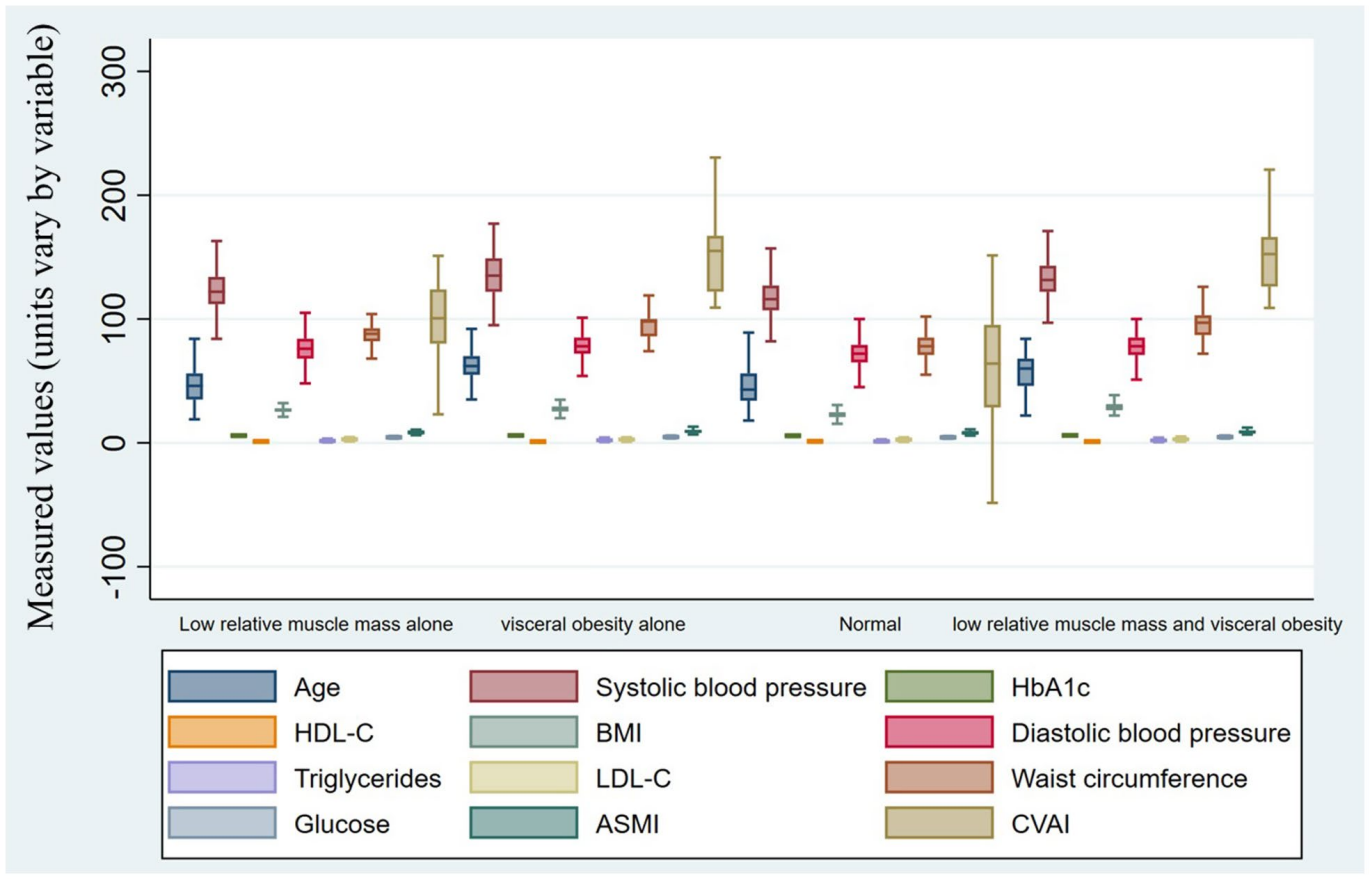
Metabolic and body-composition profiles across phenotypes. Box plots show the distributions of anthropometric, metabolic, and body-composition variables across the four body-composition phenotypes. The y-axis represents observed values, with units varying by variable. Units are as follows: age, years; systolic blood pressure and diastolic blood pressure, mmHg; BMI, kg/m^2^; waist circumference, cm; HDL-C, LDL-C, triglycerides, and fasting plasma glucose, mmol/L; HbA1c, %; ASMI, kg/m²; and CVAI, unitless index.

Compared to the normal phenotype, individuals with the low relative muscle mass alone, visceral obesity alone, and combined low relative muscle mass with visceral obesity phenotypes generally had higher body weight, BMI, waist circumference, and WHtR, with the highest adiposity-related levels observed in the combined phenotype (Table 2). Participants with visceral obesity alone tended to be older than those in the other phenotypes. Overall, omnibus tests were significant across the four phenotypes for all variables shown in Table 2. However, *post-hoc* pairwise comparisons indicated that not all six between-group contrasts were significant for every variable. The differences were most consistent for adiposity-related measures (such as BMI, waist circumference, WHtR, and the CVAI) and relative muscle mass (ASM/Wt), whereas for several metabolic variables, including SBP, DBP, and FPG, the primary distinction was observed between the normal phenotype and the three non-normal phenotypes, rather than among the non-normal phenotypes themselves. Although ASMI and absolute ASM were highest in the visceral obesity alone and combined phenotype groups, the ASM/Wt ratio was lowest in the combined phenotype group, indicating a lower muscle proportion relative to body weight (Figure 3).

**Table 2:** Anthropometric and metabolic characteristics across body composition phenotypes with omnibus group comparisons.

Variable	Normal (*n* = 2,657)	Low relative muscle mass alone (*n* = 269)	Visceral obesity alone (*n* = 465)	Low relative muscle mass and visceral obesity (*n* = 514)	Overall test statistic	*p*-Value
Age, years	45.85 ± 14.86	48.66 ± 14.84	50.82 ± 14.62	46.12 ± 14.70	16.74	<0.001
Sex [male, *n* (%)]	1,314 (49.45)	164 (60.97)	263 (56.56)	231 (44.94)	26.13	<0.001
Height, cm	164.98 ± 7.86	163.95 ± 6.69	166.73 ± 9.37	163.81 ± 8.77	12.26	<0.001
Weight, kg	60.90 ± 10.26	68.21 ± 8.73	72.63 ± 13.28	76.05 ± 14.45	364.40	<0.001
BMI, kg/m^2^	22.41 ± 2.53	25.37 ± 1.97	26.15 ± 2.84	28.26 ± 3.45	865.85	<0.001
Waist circumference, cm	76.78 ± 8.65	83.96 ± 6.81	89.36 ± 9.78	91.54 ± 10.31	582.78	<0.001
WHtR	0.47 ± 0.05	0.51 ± 0.04	0.54 ± 0.05	0.56 ± 0.05	813.64	<0.001
SBP, mmHg	118.01 ± 15.75	122.14 ± 16.15	125.03 ± 17.96	125.30 ± 15.92	48.68	<0.001
DBP, mmHg	72.31 ± 10.11	75.43 ± 9.81	75.94 ± 11.23	76.43 ± 11.24	36.75	<0.001
FPG, mmol/L	4.40 ± 0.47	4.51 ± 0.47	4.55 ± 0.53	4.58 ± 0.54	28.03	<0.001
HbA1c, %	5.69 ± 0.30	5.75 ± 0.29	5.83 ± 0.31	5.82 ± 0.32	51.72	<0.001
Triglycerides, mmol/L	1.06 (0.77–1.50)	1.23 (0.89–1.80)	1.78 (1.26–2.48)	1.65 (1.21–2.31)	531.59	<0.001
HDL-C, mmol/L	1.30 ± 0.33	1.22 ± 0.32	1.06 ± 0.24	1.08 ± 0.23	130.43	<0.001
LDL-C, mmol/L	2.61 ± 0.72	2.84 ± 0.83	2.71 ± 0.79	2.87 ± 0.76	22.49	<0.001
CVAI	58.39 ± 40.71	87.97 ± 34.48	117.43 ± 42.60	119.88 ± 44.53	523.56	<0.001
ASMI, kg/m^2^	7.96 ± 0.97	8.19 ± 0.78	8.96 ± 1.24	8.82 ± 1.15	198.86	<0.001
ASM, kg	21.86 ± 4.15	22.14 ± 3.38	25.20 ± 5.54	23.93 ± 5.07	94.79	<0.001
ASM/Wt, %	35.84 ± 2.44	32.36 ± 1.37	34.51 ± 2.01	31.36 ± 1.70	697.46	<0.001

BMI, body mass index; WHtR, waist-to-height ratio; CVAI, Chinese visceral adiposity index; ASM, appendicular skeletal muscle mass; ASMI, appendicular skeletal muscle mass index; SBP, systolic blood pressure; DBP, diastolic blood pressure; FPG, fasting plasma glucose. Values are presented as mean ± SD for approximately normally distributed variables and median (IQR) for triglycerides. Omnibus group differences were assessed using one-way ANOVA for approximately normally distributed continuous variables, the Kruskal–Wallis test for triglycerides, and the *χ*^2^ test for categorical variables. When the omnibus test was significant, *post-hoc* pairwise comparisons were performed. Detailed *post-hoc* pairwise results are provided in Supplementary Table S3.

### Prevalence of prediabetes phenotypes and logistic regression analyses

The prevalence of IFG, elevated HbA1c levels, and composite prediabetes differed across phenotypes and increased in a graded manner (*p* for trend <0.001 for each outcome). Similar overall group differences were observed for continuous glycemic measures and triglycerides, as shown in Table 2; however, *post-hoc* comparisons showed that not all pairwise contrasts were significant.

Table 3 summarizes the associations between body composition phenotypes and prediabetes outcomes across the three sequential logistic regression models. For IFG, compared to the normal phenotype, low relative muscle mass alone, visceral obesity alone, and the combined low relative muscle mass with visceral obesity phenotypes were associated with higher odds ratios in Model 1 (ORs 1.82, 2.25, and 2.66, respectively). After adjusting for age and sex (Model 2), only the combined phenotype remained associated with IFG (OR 2.84, 95% CI 1.67–4.82), while the associations for low relative muscle mass alone and visceral obesity alone phenotypes were attenuated and no longer statistically significant.

**Table 3:** Associations between body composition phenotypes and impaired fasting glucose, elevated HbA1c, and composite prediabetes.

Glucose metabolism status	Body composition phenotype	No. with abnormality	Model 1 OR (95% CI)	*p*-Value	Model 2 OR (95% CI)	*p*-Value	Model 3 OR (95% CI)	*p*-Value
Impaired fasting glucose (IFG)	Normal (*n* = 2,657)	44	1.00 (reference)	–	1.00 (reference)	–	1.00 (reference)	–
	Low relative muscle mass alone (*n* = 269)	8	1.82 (0.85–3.91)	0.124	1.52 (0.70–3.30)	0.288	1.57 (0.73–3.41)	0.248
	Visceral obesity alone (*n* = 465)	17	2.25 (1.28–3.98)	0.005	1.75 (0.98–3.11)	0.059	1.51 (0.86–2.67)	0.149
	Low relative muscle mass and visceral obesity (*n* = 514)	22	2.66 (1.58–4.47)	<0.001	2.84 (1.67–4.82)	<0.001	2.45 (1.32–4.55)	0.004
Elevated HbA1c	Normal (*n* = 2,657)	1,419	1.00 (reference)	–	1.00 (reference)	–	1.00 (reference)	–
	Low relative muscle mass alone (*n* = 269)	167	1.43 (1.10–1.85)	0.007	1.22 (0.92–1.62)	0.163	1.12 (0.83–1.52)	0.441
	Visceral obesity alone (*n* = 465)	324	2.00 (1.62–2.48)	<0.001	1.64 (1.30–2.06)	<0.001	1.43 (1.12–1.82)	0.004
	Low relative muscle mass and visceral obesity (*n* = 514)	365	2.14 (1.74–2.62)	<0.001	2.42 (1.95–3.02)	<0.001	2.06 (1.61–2.63)	<0.001
Combined prediabetes (PDM: IFG or elevated HbA1c)	Normal (*n* = 2,657)	1,423	1.00 (reference)	–	1.00 (reference)	–	1.00 (reference)	–
	Low relative muscle mass alone (*n* = 269)	168	1.44 (1.11–1.87)	0.005	1.24 (0.93–1.64)	0.142	1.14 (0.85–1.54)	0.380
	Visceral obesity alone (*n* = 465)	325	2.01 (1.63–2.49)	<0.001	1.64 (1.31–2.07)	<0.001	1.42 (1.11–1.81)	0.005
	Low relative muscle mass and visceral obesity (*n* = 514)	366	2.14 (1.75–2.63)	<0.001	2.44 (1.95–3.04)	<0.001	2.07 (1.62–2.65)	<0.001

Pseudo *R*^2^: IFG: Model 1 = 0.0189; Model 2 = 0.0939; Model 3 = 0.1113; Elevated HbA1c: Model 1 = 0.0167; Model 2 = 0.1377; Model 3 = 0.1506. Combined prediabetes: Model 1 = 0.0169; Model 2 = 0.1385; Model 3 = 0.1506. Model 1 was unadjusted. Model 2 was adjusted for age and sex. Model 3 was further adjusted for systolic blood pressure, diastolic blood pressure, LDL-C, ALT, and eGFR. Model 3 was based on a complete-case sample (*n* = 3,567) because of missing covariate data. ORs are presented with 95% confidence intervals. Pseudo *R*^2^ values are McFadden’s pseudo *R*^2^.

For elevated HbA1c levels, all three non-normal phenotypes were associated with higher odds ratios in Model 1. In Model 2, visceral obesity alone (OR 1.64, 95% CI 1.30–2.06) and combined low relative muscle mass with visceral obesity (OR 2.42, 95% CI 1.95–3.02) phenotypes remained associated with elevated HbA1c levels, whereas low relative muscle mass alone was not significant after adjustment (Table 3).

For composite prediabetes, visceral obesity alone (OR 1.64, 95% CI 1.31–2.07) and combined low relative muscle mass with visceral obesity (OR 2.44, 95% CI 1.95–3.04) phenotypes remained associated after adjusting for age and sex, whereas low relative muscle mass alone was not significant (Table 3).

In the extended confounder-adjusted model (Model 3), the phenotype combining visceral obesity and low relative muscle mass remained independently associated with IFG (OR 2.45, 95% CI 1.32–4.55), elevated HbA1c levels (OR 2.06, 95% CI 1.61–2.63), and composite prediabetes (OR 2.07, 95% CI 1.62–2.65). Visceral obesity alone remained associated with elevated HbA1c levels and composite prediabetes, whereas low relative muscle mass alone was not independently associated with any outcome.

## Discussion

Overall, our analysis shows a CVAI-defined phenotype that combines visceral obesity and low relative muscle mass, which is the body composition phenotype most strongly associated with dysglycemia. Compared to the reference phenotype, the combined low relative muscle mass and visceral obesity phenotype showed the highest adjusted odds ratio across prediabetes outcomes, whereas visceral obesity alone was more consistently linked to HbA1c-defined dysglycemia and the composite prediabetes definition. In addition, the CVAI demonstrated the best discriminative performance among the adiposity indicators evaluated. Taken together, these findings suggest that visceral adiposity and reduced muscle reserve jointly shape early disturbances in glucose metabolism and that a visceral fat-focused metric may better capture risk than general adiposity measures in this population.

Placing these findings in the context of prior research, our data support a synergistic effect between adiposity and low muscle mass on metabolic risk (8, 15–17, 28, 32). The weaker association observed for low relative muscle mass alone after multivariable adjustment may reflect confounding by age and sex (33) and the fact that early dysglycemia in generally healthy adults is often driven more strongly by excess adiposity than by low muscle mass (34). Mechanistically, visceral fat promotes insulin resistance through ectopic lipid deposition, chronic low-grade inflammation, and adverse adipokine profiles, while reduced muscle mass diminishes the primary tissue reservoir for insulin-mediated glucose disposal (8, 9, 13). The combination of high visceral fat and low muscle reserve may therefore exacerbate insulin resistance and impair glucose homeostasis beyond the effects of either component alone (13, 14).

Our results also emphasize that the choice of adiposity metric is important. While BMI and simple waist measures are convenient, they cannot distinguish visceral fat from subcutaneous fat and may misclassify individuals with normal-weight central adiposity or those with substantial visceral fat despite a relatively modest BMI (20, 21). The CVAI was developed for Chinese adults and integrates age and lipid parameters that correlate with visceral fat and metabolic dysfunction, which may help explain its stronger associations and better discrimination (20). However, the CVAI incorporates age, triglycerides, and HDL-C, all of which are closely related to glycemic status. Therefore, the superior AUC of the CVAI compared to BMI and WHtR should be interpreted with caution, as it may partly reflect the inclusion of additional metabolic parameters rather than a purely superior ability to capture visceral fat. In addition, age is included both in the CVAI formula and as an adjustment and stratification variable in our models, which may further complicate causal interpretation. Taken together, our ROC analyses are better viewed as evidence for improved risk stratification rather than proof that the CVAI is intrinsically superior to simpler anthropometric indices.

For the muscle component, it is important to interpret the phenotype labels in light of how low relative muscle mass was operationalized. Individuals classified as having low relative muscle mass by ASM/Wt could still exhibit higher absolute ASM or ASMI, as larger body size inflates absolute lean mass while lowering the proportion of muscle relative to total body mass (35, 36). From a metabolic perspective, the muscle-to-weight ratio may better capture the mismatch between glucose-handling capacity and body size (37). Nonetheless, muscle impairment is multidimensional, and current consensus definitions of sarcopenia require evidence of low muscle strength and/or impaired physical performance in addition to reduced muscle mass. Our mass-based proxy should therefore not be equated with clinically diagnosed sarcopenia, and future studies should incorporate muscle strength, physical performance, and muscle quality to refine phenotyping.

Several strengths support the credibility of these observations, including the relatively large sample from a routine health check-up setting, standardized assessments, and the concurrent evaluation of multiple prediabetes phenotypes. Important limitations should also be noted. The cross-sectional design precludes causal inference and raises the possibility of reverse causation. Prediabetes was defined using fasting glucose and HbA1c levels; without an oral glucose tolerance test, we could not evaluate impaired glucose tolerance, and some cases may have been missed (38). Body composition was assessed using BIA, a method that is less precise than reference methods and may introduce measurement errors (39). In addition, the extended confounder-adjusted model was based on complete-case analysis due to missing covariate data, which may have introduced selection bias if the missing data were not completely random. Moreover, while age- and sex-specific percentile cutoffs were used and adjusted for sex was in the regression models, we did not formally assess sex-specific interactions or conduct sex-stratified analyses; therefore, the potential sex differences in the observed associations warrant further investigation. Residual confounding factors, such as dietary patterns, medication use, and inflammatory markers, and the single-center setting may also limit the generalizability of the findings.

These findings have practical implications for early prevention. Screening approaches that consider both visceral adiposity and muscle reserve may better identify high-risk individuals than reliance on BMI alone. In routine health check-up settings, combining the CVAI with a simple muscle indicator could serve as a low-cost triage tool to prompt confirmatory testing and targeted counselling. Intervention strategies may also need to be dual-focused, reducing visceral fat through lifestyle modification while preserving or increasing muscle reserve through resistance training and adequate dietary protein intake (40, 41). Prospective studies and intervention trials are needed to determine whether reducing visceral adiposity while improving relative muscle reserve can meaningfully reduce progression from prediabetes to diabetes.

## Conclusion

In this large cross-sectional study of Chinese adults who underwent health check-ups, the phenotype combining low ASM/Wt and high CVAI-defined visceral adiposity was independently and more strongly associated with prediabetes outcomes than either low relative muscle mass alone or visceral obesity alone. The prevalence and odds ratio of IFG, elevated HbA1c levels, and composite prediabetes increased in a graded manner across body composition phenotypes, with the highest values consistently observed in the combined phenotype. Visceral obesity alone remained independently associated with elevated HbA1c levels and composite prediabetes, whereas the associations for low relative muscle mass alone were attenuated after adjusting for age and sex. The CVAI outperformed both BMI and the WHtR in discriminating abnormal glycemia, highlighting the potential importance of visceral adiposity in early dysglycemia among Chinese adults. These findings support combining muscle mass and visceral fat assessments for prediabetes screening and risk stratification and provide a rationale for targeting coexisting low relative muscle mass and visceral obesity in prevention strategies.

## Data Availability

The raw data supporting the conclusions of this article will be made available by the authors, without undue reservation.

## References

[1] Rooney MR He JH Salpea P Genitsaridi I Magliano DJ Boyko EJ . Global and regional prediabetes prevalence: updates for 2024 and projections for 2050. Diabetes Care. (2025) 48:e142–4. doi: 10.2337/dc25-1640, 40925012 PMC12435923

[2] Li Y Teng D Shi X Qin G Qin Y Quan H . Prevalence of diabetes recorded in mainland China using 2018 diagnostic criteria from the American Diabetes Association: national cross sectional study. BMJ. (2020) 369:m997. doi: 10.1136/bmj.m997 32345662 PMC7186854

[3] Wang L Gao P Zhang M Huang Z Zhang D Deng Q . Prevalence and ethnic pattern of diabetes and prediabetes in China in 2013. JAMA. (2017) 317:2515–23. doi: 10.1001/jama.2017.7596 28655017 PMC5815077

[4] Wang L Peng W Zhao Z Zhang M Shi Z Song Z . Prevalence and treatment of diabetes in China, 2013–2018. JAMA. (2021) 326:2498–506. doi: 10.1001/jama.2021.22208, 34962526 PMC8715349

[5] Yu X Duan F Lin D Li H Zhang J Wang Q . Prevalence of diabetes, prediabetes, and associated factors in an adult Chinese population: baseline of a prediabetes cohort study. Int J Endocrinol. (2020) 2020:1–8. doi: 10.1155/2020/8892176 PMC7707990 33299413

[6] Wang R Zhang P Li Z Lv X Cai H Gao C . The prevalence of pre-diabetes and diabetes and their associated factors in Northeast China: a cross-sectional study. Sci Rep. (2019) 9:2513. doi: 10.1038/s41598-019-39221-2, 30792436 PMC6385189

[7] Ma RCW. Epidemiology of diabetes and diabetic complications in China. Diabetologia. (2018) 61:1249–60. doi: 10.1007/s00125-018-4557-7, 29392352

[8] Hong SH Choi KM. Sarcopenic obesity, insulin resistance, and their implications in cardiovascular and metabolic consequences. Int J Mol Sci. (2020) 21:494. doi: 10.3390/ijms21020494 31941015 PMC7013734

[9] Wang M Tan Y Shi Y Wang X Liao Z Wei P. Diabetes and sarcopenic obesity: pathogenesis, diagnosis, and treatments. Front Endocrinol (Lausanne). (2020) 11:568. doi: 10.3389/fendo.2020.00568, 32982969 PMC7477770

[10] Correa-de-Araujo R Addison O Miljkovic I Goodpaster BH Bergman BC Clark RV . Myosteatosis in the context of skeletal muscle function deficit: an interdisciplinary workshop at the National Institute on aging. Front Physiol. (2020) 11:963. doi: 10.3389/fphys.2020.00963, 32903666 PMC7438777

[11] Kolb H. Obese visceral fat tissue inflammation: from protective to detrimental? BMC Med. (2022) 20:494. doi: 10.1186/s12916-022-02672-y, 36575472 PMC9795790

[12] Wei S Nguyen TT Zhang Y Ryu D Gariani K. Sarcopenic obesity: epidemiology, pathophysiology, cardiovascular disease, mortality, and management. Front Endocrinol (Lausanne). (2023) 14:1185221. doi: 10.3389/fendo.2023.1185221, 37455897 PMC10344359

[13] Donini LM Busetto L Bischoff SC Cederholm T Ballesteros-Pomar MD Batsis JA . Definition and diagnostic criteria for sarcopenic obesity: ESPEN and EASO consensus statement. Obes Facts. (2022) 15:321–35. doi: 10.1159/000521241 35196654 PMC9210010

[14] Cawthon PM Peters KW Shardell MD McLean RR Dam TT Kenny AM . Cutpoints for low appendicular lean mass that identify older adults with clinically significant weakness. J Gerontol A Biol Sci Med Sci. (2014) 69:567–75. doi: 10.1093/gerona/glu023, 24737559 PMC3991141

[15] Qiao Y-S Chai Y-H Gong H-J Zhuldyz Z Stehouwer CDA Zhou J-B . The association between diabetes mellitus and risk of sarcopenia: accumulated evidences from observational studies. Front Endocrinol. (2021) 12:782391. doi: 10.3389/fendo.2021.782391, 35002965 PMC8734040

[16] Yuan J Jia P. Prediabetes and diabetes were attributed to the prevalence and severity of sarcopenia in middle-aged and elderly adults. Diabetol Metab Syndr. (2024) 16:122. doi: 10.1186/s13098-024-01355-3, 38825679 PMC11145839

[17] Xu J Han X Chen Q Cai M Tian J Yan Z . Association between sarcopenia and prediabetes among non-elderly US adults. J Endocrinol Investig. (2023) 46:1815–24. doi: 10.1007/s40618-023-02038-y 36856982

[18] Donini LM Busetto L Bauer JM Bischoff S Boirie Y Cederholm T . Critical appraisal of definitions and diagnostic criteria for sarcopenic obesity based on a systematic review. Clin Nutr. (2020) 39:2368–88. doi: 10.1016/j.clnu.2019.11.024 31813698

[19] Chen TP Kao HH Ogawa W Arai H Tahapary DL Assantachai P . The Asia–Oceania consensus: definitions and diagnostic criteria for sarcopenic obesity. Obes Res Clin Pract. (2025) 19:185–92. doi: 10.1016/j.orcp.2025.05.001, 40335420

[20] Xia MF Chen Y Lin HD Ma H Li XM Aleteng Q . A indicator of visceral adipose dysfunction to evaluate metabolic health in adult Chinese. Sci Rep. (2016) 6:38214. doi: 10.1038/srep38214, 27905531 PMC5131270

[21] Börgeson E Tavajoh S Lange S Jessen N. The challenges of assessing adiposity in a clinical setting. Nat Rev Endocrinol. (2024) 20:615–26. doi: 10.1038/s41574-024-01012-9, 39009863

[22] Khadra D Itani L Tannir H Kreidieh D El Masri D El Ghoch M. Association between sarcopenic obesity and higher risk of type 2 diabetes in adults: a systematic review and meta-analysis. World J Diabetes. (2019) 10:311–23. doi: 10.4239/wjd.v10.i5.311, 31139318 PMC6522758

[23] Wu J Gong L Li Q Hu J Zhang S Wang Y . A novel visceral adiposity index for prediction of type 2 diabetes and pre-diabetes in Chinese adults: a 5-year prospective study. Sci Rep. (2017) 7:13784. doi: 10.1038/s41598-017-14251-w, 29062099 PMC5653832

[24] Lee DY. The prevalence of and factors associated with Sarcopenic obesity, sarcopenia, and obesity among Korean adults: findings from the 2022–2023 Korea National Health and nutrition examination survey. Medicina (Kaunas). (2025) 61:1424. doi: 10.3390/medicina61081424 40870469 PMC12388213

[25] Zamboni M Rubele S Rossi AP. Sarcopenia and obesity. Curr Opin Clin Nutr Metab Care. (2019) 22:13–9. doi: 10.1097/MCO.0000000000000519, 30461451

[26] Liu C Cheng KY Tong X Cheung WH Chow SK Law SW . The role of obesity in sarcopenia and the optimal body composition to prevent against sarcopenia and obesity. Front Endocrinol (Lausanne). (2023) 14:1077255. doi: 10.3389/fendo.2023.1077255, 36936175 PMC10016224

[27] Jang IY Jung HW Lee CK Yu SS Lee YS Lee E. Comparisons of predictive values of sarcopenia with different muscle mass indices in Korean rural older adults: a longitudinal analysis of the aging study of PyeongChang rural area. Clin Interv Aging. (2018) 13:91–9. doi: 10.2147/CIA.S155619 29391783 PMC5769584

[28] Srikanthan P Karlamangla AS. Relative muscle mass is inversely associated with insulin resistance and prediabetes. Findings from the third National Health and nutrition examination survey. J Clin Endocrinol Metabol. (2011) 96:2898–903. doi: 10.1210/jc.2011-0435, 21778224

[29] Hong S Chang Y Jung HS Yun KE Shin H Ryu S. Relative muscle mass and the risk of incident type 2 diabetes: a cohort study. PLoS One. (2017) 12:e0188650. doi: 10.1371/journal.pone.0188650, 29190709 PMC5708784

[30] Han M Qie R Li Q Liu L Huang S Wu X . Chinese visceral adiposity index, a novel indicator of visceral obesity for assessing the risk of incident hypertension in a prospective cohort study. Br J Nutr. (2021) 126:612–20. doi: 10.1017/S0007114520004298, 33143773

[31] Ye X Zhang G Han C Wang P Lu J Zhang M. The association between Chinese visceral adiposity index and cardiometabolic multimorbidity among Chinese middle-aged and older adults: a national cohort study. Front Endocrinol (Lausanne). (2024) 15:1381949. doi: 10.3389/fendo.2024.1381949, 38601202 PMC11004471

[32] Cheng G Zhou Y Wang Y Tao L Xu J. The relationship between sarcopenic obesity and prediabetes in adolescents: analysis of the national health and nutrition examination survey from 2011 to 2016. J Clin Transl Endocrinol. (2025) 41:100414. doi: 10.1016/j.jcte.2025.100414 40978534 PMC12447563

[33] Cruz-Jentoft AJ Bahat G Bauer J Boirie Y Bruyère O Cederholm T . Sarcopenia: revised European consensus on definition and diagnosis. Age Ageing. (2019) 48:16–31. doi: 10.1093/ageing/afy169, 30312372 PMC6322506

[34] Vera-Ponce VJ Zuzunaga-Montoya FE Vásquez-Romero LEM Loayza-Castro JA Iturregui Paucar CR Gutiérrez De Carrillo CI . Anthropometric measures of obesity as risk indicators for prediabetes. A systematic review and meta-analysis. Diabet Epidemiol Manag. (2024) 16:100230. doi: 10.1016/j.deman.2024.100230

[35] Kim KM Jang HC Lim S. Differences among skeletal muscle mass indices derived from height-, weight-, and body mass index-adjusted models in assessing sarcopenia. Korean J Intern Med. (2016) 31:643–50. doi: 10.3904/kjim.2016.015 27334763 PMC4939509

[36] Kim TN Park MS Lee EJ Chung HS Yoo HJ Kang HJ . Comparisons of three different methods for defining sarcopenia: an aspect of cardiometabolic risk. Sci Rep. (2017) 7:6491. doi: 10.1038/s41598-017-06831-7, 28747657 PMC5529503

[37] Prado CM Batsis JA Donini LM Gonzalez MC Siervo M. Sarcopenic obesity in older adults: a clinical overview. Nat Rev Endocrinol. (2024) 20:261–77. doi: 10.1038/s41574-023-00943-z, 38321142 PMC12854800

[38] American Diabetes Association Professional Practice Committee. 2. Diagnosis and classification of diabetes: standards of Care in Diabetes—2024. Diabetes Care. (2024) 47:S20–42. doi: 10.2337/dc24-S002 38078589 PMC10725812

[39] Kim Y Beom J Lee SY Jang HC Kim K Kim M . Comparison of bioelectrical impedance analysis and dual-energy X-ray absorptiometry for the diagnosis of sarcopenia in the older adults with metabolic syndrome: equipment-specific equation development. Aging Clin Exp Res. (2024) 37:12. doi: 10.1007/s40520-024-02898-1, 39725814 PMC11671549

[40] Zhang H Guo Y Hua G Guo C Gong S Li M . Exercise training modalities in prediabetes: a systematic review and network meta-analysis. Front Endocrinol (Lausanne). (2024) 15:1308959. doi: 10.3389/fendo.2024.1308959, 38440785 PMC10911289

[41] Nishimura Y Højfeldt G Breen L Tetens I Holm L. Dietary protein requirements and recommendations for healthy older adults: a critical narrative review of the scientific evidence. Nutr Res Rev. (2023) 36:69–85. doi: 10.1017/S0954422421000329, 34666855

