# Supplementary material for: Association between CVAI-defined body composition phenotypes and prediabetes outcomes in Chinese adults undergoing health check-ups: a cross-sectional study
Source: Front Nutr. 2026 May 14;13:1784292. doi: 10.3389/fnut.2026.1784292 (PMC13216018; doi:10.3389/fnut.2026.1784292)
Supplement: Supplementary file 1 [file Supplementary_file_1.docx]

**Supplementary Files**

[**Table S1. Laboratory assays, instruments, and quality-control procedures** 2](#_Toc227083537)

[**Table S2. Sex- and age-stratified cut-offs for low relative muscle mass and visceral obesity** 3](#_Toc227083538)

**Table S3. Pairwise post-hoc comparisons for variables included in Table 2** [4](#_Toc227083538)

**Table S1. Laboratory assays, instruments, and quality-control procedures**

| Domain | Biomarker / measurement | Specimen | Method principle | Analyzer (model) | Manufacturer | Unit |
| --- | --- | --- | --- | --- | --- | --- |
| Glycemia | FPG | plasma | hexokinase | ADVIA 2400 / Chemistry XPT | Siemens | mmol/L |
| Glycemia | HbA1c | whole blood | HPLC | Premier Hb9210 | Trinity Biotech | % |
| Lipids | TG | serum | enzymatic | ADVIA 2400 / Chemistry XPT | Siemens | mmol/L |
| Lipids | HDL-C | serum | direct enzymatic | ADVIA 2400 / Chemistry XPT | Siemens | mmol/L |
| Lipids | LDL-C | serum | direct/ calculated | ADVIA 2400 / Chemistry XPT | Siemens | mmol/L |
| Anthropometrics | BP | - | oscillometric | BP-203RPE III | Omron | mmHg |
| Body composition | BIA | - | multifrequency segmental | X-SCAN PLUS | ACCUNIQ |  |

**Table S2. Sex- and age-stratified cut-offs for low relative muscle mass and visceral obesity**

| Sex | Age stratum (years) | N | ASM/Wt P20 | CVAI P75 | Notes |
| --- | --- | --- | --- | --- | --- |
| Female | 18-39 | 922 | 0.32 | 35.95 | Sample-derived percentiles |
| Female | 40-59 | 705 | 0.31 | 84.51 | Sample-derived percentiles |
| Female | ≥60 | 306 | 0.30 | 122.42 | Sample-derived percentiles |
| Male | 18-39 | 588 | 0.34 | 109.08 | Sample-derived percentiles |
| Male | 40-59 | 805 | 0.34 | 126.54 | Sample-derived percentiles |
| Male | ≥60 | 579 | 0.34 | 139.49 | Sample-derived percentiles |

**Table S3. Pairwise post-hoc comparisons for variables included in Table 2**

| Variable | Post-hoc method | Normal vs Low relative muscle mass alone | Normal vs visceral obesity alone | Normal vs low relative muscle mass and visceral obesity | Low relative muscle mass alone vs visceral obesity alone | Low relative muscle mass alone vs low relative muscle mass and visceral obesity | visceral obesity alone vs low relative muscle mass and visceral obesity |
| --- | --- | --- | --- | --- | --- | --- | --- |
| Age, years | Bonferroni-adjusted pairwise comparison after one-way ANOVA | 0.018 | 0.000 | 1.000 | 0.342 | 0.136 | 0.000 |
| Sex [male, n (%)] | Pairwise χ² tests with Bonferroni adjustment | 0.0018 | 0.0282 | 0.3660 | 1.000 | 0.0000 | 0.0018 |
| Height, cm | Bonferroni-adjusted pairwise comparison after one-way ANOVA | 0.279 | 0.000 | 0.016 | 0.000 | 1.000 | 0.000 |
| Weight, kg | Bonferroni-adjusted pairwise comparison after one-way ANOVA | 0.000 | 0.000 | 0.000 | 0.000 | 0.000 | 0.000 |
| BMI, kg/m² | Bonferroni-adjusted pairwise comparison after one-way ANOVA | 0.000 | 0.000 | 0.000 | 0.001 | 0.000 | 0.000 |
| Waist circumference, cm | Bonferroni-adjusted pairwise comparison after one-way ANOVA | 0.000 | 0.000 | 0.000 | 0.000 | 0.000 | 0.001 |
| WHtR | Bonferroni-adjusted pairwise comparison after one-way ANOVA | 0.000 | 0.000 | 0.000 | 0.000 | 0.000 | 0.000 |
| SBP, mmHg | Bonferroni-adjusted pairwise comparison after one-way ANOVA | 0.000 | 0.000 | 0.000 | 0.114 | 0.054 | 1.000 |
| DBP, mmHg | Bonferroni-adjusted pairwise comparison after one-way ANOVA | 0.000 | 0.000 | 0.000 | 1.000 | 1.000 | 1.000 |
| FPG, mmol/L | Bonferroni-adjusted pairwise comparison after one-way ANOVA | 0.004 | 0.000 | 0.000 | 1.000 | 0.311 | 1.000 |
| HbA1c, % | Bonferroni-adjusted pairwise comparison after one-way ANOVA | 0.006 | 0.000 | 0.000 | 0.003 | 0.009 | 1.000 |
| Triglycerides, mmol/L | Pairwise Mann-Whitney U tests with Bonferroni adjustment | 0.0000 | 0.0000 | 0.0000 | 0.0000 | 0.0000 | 0.2142 |
| HDL-C, mmol/L | Bonferroni-adjusted pairwise comparison after one-way ANOVA | 0.001 | 0.000 | 0.000 | 0.000 | 0.000 | 1.000 |
| LDL-C, mmol/L | Bonferroni-adjusted pairwise comparison after one-way ANOVA | 0.000 | 0.045 | 0.000 | 0.150 | 1.000 | 0.007 |
| CVAI | Bonferroni-adjusted pairwise comparison after one-way ANOVA | 0.000 | 0.000 | 0.000 | 0.000 | 0.000 | 1.000 |
| ASMI, kg/m² | Bonferroni-adjusted pairwise comparison after one-way ANOVA | 0.004 | 0.000 | 0.000 | 0.000 | 0.000 | 0.180 |
| ASM, kg | Bonferroni-adjusted pairwise comparison after one-way ANOVA | 1.000 | 0.000 | 0.000 | 0.000 | 0.000 | 0.000 |
| ASM/Wt, % | Bonferroni-adjusted pairwise comparison after one-way ANOVA | 0.000 | 0.000 | 0.000 | 0.000 | 0.000 | 0.000 |

Supplementary Table S3. Pairwise post-hoc comparisons for variables included in Table 2. For approximately normally distributed continuous variables, Bonferroni-adjusted pairwise comparisons following one-way ANOVA were used. For triglycerides, pairwise Mann-Whitney rank-sum tests were performed with Bonferroni adjustment for six comparisons. For sex, pairwise χ² tests of proportions were performed with Bonferroni adjustment.
