# Supplementary material for: Association between CVAI-defined body composition phenotypes and prediabetes outcomes in Chinese adults undergoing health check-ups: a cross-sectional study
Source: Front Nutr. 2026 May 14;13:1784292. doi: 10.3389/fnut.2026.1784292 (PMC13216018; doi:10.3389/fnut.2026.1784292)
Supplement: Supplementary file 2 [file Supplementary_file_2.docx]

STROBE Statement—Checklist of items that should be included in reports of ***cross-sectional studies***

|  | Item No | Recommendation | Location in manuscript |
| --- | --- | --- | --- |
| **Title and abstract** | 1 | (*a*) Indicate the study’s design with a commonly used term in the title or the abstract | Title; Abstract |
|  |  | (*b*) Provide in the abstract an informative and balanced summary of what was done and what was found | Abstract |
| Introduction | | | |
| Background/rationale | 2 | Explain the scientific background and rationale for the investigation being reported | Introduction |
| Objectives | 3 | State specific objectives, including any prespecified hypotheses | Introduction - final paragraph |
| Methods | | | |
| Study design | 4 | Present key elements of study design early in the paper | Methods - Study design and population |
| Setting | 5 | Describe the setting, locations, and relevant dates, including periods of recruitment, exposure, follow-up, and data collection | Methods - Study design and population |
| Participants | 6 | (*a*) Give the eligibility criteria, and the sources and methods of selection of participants | Methods - Study design and population; Figure 1 |
| Variables | 7 | Clearly define all outcomes, exposures, predictors, potential confounders, and effect modifiers. Give diagnostic criteria, if applicable | Methods - outcomes, phenotypes, covariates, and statistical analysis |
| Data sources/ measurement | 8* | For each variable of interest, give sources of data and details of methods of assessment (measurement). Describe comparability of assessment methods if there is more than one group | Methods - measurements; Supplementary Table S1 |
| Bias | 9 | Describe any efforts to address potential sources of bias | Discussion - limitations |
| Study size | 10 | Explain how the study size was arrived at | Methods - Study design and population |
| Quantitative variables | 11 | Explain how quantitative variables were handled in the analyses. If applicable, describe which groupings were chosen and why | Methods - phenotype definitions and statistical analysis; Supplementary Table S2 |
| Statistical methods | 12 | (*a*) Describe all statistical methods, including those used to control for confounding | Methods - Statistical analysis |
|  |  | (*b*) Describe any methods used to examine subgroups and interactions | Not applicable - no subgroup or interaction analyses |
|  |  | (*c*) Explain how missing data were addressed | Methods - Study design and population; Statistical analysis |
|  |  | (*d*) If applicable, describe analytical methods taking account of sampling strategy | Not applicable - no sampling strategy |
|  |  | (*e*) Describe any sensitivity analyses | Not applicable - no sensitivity analyses |
| Results | | | |
| Participants | 13* | (a) Report numbers of individuals at each stage of study—eg numbers potentially eligible, examined for eligibility, confirmed eligible, included in the study, completing follow-up, and analysed | Methods - Study design and population; Results - Participant characteristics; Figure 1 |
|  |  | (b) Give reasons for non-participation at each stage | Methods - Study design and population; Figure 1 |
|  |  | (c) Consider use of a flow diagram | Figure 1 |
| Descriptive data | 14* | (a) Give characteristics of study participants (eg demographic, clinical, social) and information on exposures and potential confounders | Results - Participant characteristics; Table 2 |
|  |  | (b) Indicate number of participants with missing data for each variable of interest | Figure 1; Methods - Statistical analysis |
| Outcome data | 15* | Report numbers of outcome events or summary measures | Results - Participant characteristics; Table 3 |
| Main results | 16 | (*a*) Give unadjusted estimates and, if applicable, confounder-adjusted estimates and their precision (eg, 95% confidence interval). Make clear which confounders were adjusted for and why they were included | Results - logistic regression analyses; Table 3 |
|  |  | (*b*) Report category boundaries when continuous variables were categorized | Methods - phenotype definitions; Supplementary Table S2 |
|  |  | (*c*) If relevant, consider translating estimates of relative risk into absolute risk for a meaningful time period | Not applicable - cross-sectional study |
| Other analyses | 17 | Report other analyses done—eg analyses of subgroups and interactions, and sensitivity analyses | Results - ROC analyses; Table 1; Figure 2 |
| Discussion | | | |
| Key results | 18 | Summarise key results with reference to study objectives | Discussion - first paragraph |
| Limitations | 19 | Discuss limitations of the study, taking into account sources of potential bias or imprecision. Discuss both direction and magnitude of any potential bias | Discussion - limitations paragraph |
| Interpretation | 20 | Give a cautious overall interpretation of results considering objectives, limitations, multiplicity of analyses, results from similar studies, and other relevant evidence | Discussion - interpretation paragraphs |
| Generalisability | 21 | Discuss the generalisability (external validity) of the study results | Discussion - limitations paragraph |
| Other information | | | |
| Funding | 22 | Give the source of funding and the role of the funders for the present study and, if applicable, for the original study on which the present article is based | Declarations - Funding |

*Give information separately for exposed and unexposed groups.

**Note:** An Explanation and Elaboration article discusses each checklist item and gives methodological background and published examples of transparent reporting. The STROBE checklist is best used in conjunction with this article (freely available on the Web sites of PLoS Medicine at http://www.plosmedicine.org/, Annals of Internal Medicine at http://www.annals.org/, and Epidemiology at http://www.epidem.com/). Information on the STROBE Initiative is available at www.strobe-statement.org.
